# Health professionals who have worked in COVID-19 immunization centers suffer the effects of violence

**DOI:** 10.3389/fpubh.2023.1264301

**Published:** 2023-09-20

**Authors:** Laura Brunelli, Enrico Scarpis, Tancredi Lo Presti, Francesca Fiorillo, Fabio Campanella, Paola Zuliani, Federico Farneti, Eleonora Croci, Barbara Pellizzari, Roberto Cocconi, Luca Arnoldo

**Affiliations:** ^1^Department of Medicine, University of Udine, Udine, Italy; ^2^Clinical Risk, Quality and Accreditation Unit, Friuli Centrale Healthcare University Trust, Udine, Italy; ^3^Medical Directorate of Palmanova-Latisana Hospital, Friuli Centrale Healthcare University Trust, Udine, Italy; ^4^Regional Transplant Centre, Friuli Centrale Healthcare University Trust, Udine, Italy; ^5^Neurosurgery Unit, Friuli Centrale Healthcare University Trust, Udine, Italy; ^6^Department of Prevention, Giuliano Isontina Healthcare University Trust, Trieste, Italy; ^7^Department of Prevention, Friuli Occidentale Healthcare Trust, Pordenone, Italy

**Keywords:** violence, health professionals, impact, COVID-19 vaccination, workplace violence (WPV)

## Abstract

**Background:**

The phenomenon violence against health professionals has received increasing attention in recent years because of its frequency and significant impact on victims’ mental health and disruption of health services. Despite this attention, little is known about the incidence of workplace violence in the highly politicized immunization services. Therefore, we decided to examine the prevalence of workplace violence in the COVID-19 immunization campaign, the risk and protective factors, and the impact on victims’ mental health.

**Methods:**

Between March and April 2022, we conducted an anonymous online survey among health professionals working in COVID-19 vaccination centers in the Friuli-Venezia Giulia Region (Italy). We used the Questionnaire for Workplace Violence in Healthcare Settings and the Impact of Event Scale–Revised.

**Results:**

Of the 200 participants, 93 (46.5%) reported being victims of an act of violence during the vaccination campaign, 60 of them verbally and 7 physically. In 35.5% of cases, the IES score indicated a possible post-traumatic stress reaction in the victim. Opinions on measures to prevent violence and support workers in the workplace differed according to the sex of the health professional, with women emphasizing the need for self-defense training and improvement of security arrangements (*p* < 0.001).

**Conclusion:**

One-third of health professionals involved in the COVID-19 immunization campaign reported that their mental health was affected by workplace violence. Public health professionals dealing with politicized and debated issues such as immunization should receive more attention, as should the implementation of a more structured and multidisciplinary approach to the problem within healthcare organizations.

## Background

The World Health Organization defines workplace violence (WPV) as “incidents where staff is abused, threatened, or assaulted in circumstances related to their work, including commuting to and from work, involving an explicit or implicit challenge to their safety, wellbeing or health” (1). Workplace violence includes both physical and verbal violence and can be categorized into four types depending on the perpetrator’s relationship to the workplace. Type II violence is the case perpetrated by a patient (2).

In recent years, the WPV phenomenon has been referred to as a silent epidemic (3) that accompanies the COVID-19 pandemic that the world has known since 2020 regardless of a country’s security situation (4) or work environment, organizational culture, and access to resources (5). The burden of this problem has been studied and discussed by many authors (6–8), but it still seems to be underestimated because of a lack of systematic recording (3) and a high underreporting rate, which is partly related to resignation and the misperception of this behavior as an inherent state of frailty and powerlessness of the patient. The overall prevalence of WPV is 58.7%, with verbal violence (66.8%) predominating over physical violence, which in any case reaches a worrying level (20.8%) (9) and shows differences between professional profiles (10). In most cases, this violence is perpetrated by patients (11), which has a dramatic impact on the physical and mental health of health professionals (6). Nonetheless, the impact of the COVID-19 pandemic on the general public and health professionals has raised a number of COVID-related health issues, such as the uncertain health, economic, and, because of recent developments, political situation, which has disturbed the balance at all levels, generating stress and, at best, even leading to poor mental health status (12–15).

Despite the very early warning of the enormous pressure the pandemic would place on healthcare workers (16), and given the high level of attention given to health professionals working in emergency care and highly politicized healthcare services, which according to Kuhlmann et al. (17) include vaccination centers as well as services that provide abortion and reproductive healthcare, and services for minorities and vulnerable groups (e.g., asylum seekers, migrants, LGBTQ persons), there have been no studies, to our knowledge, that have examined the incidence of violent episodes specifically related to the COVID-19 vaccination campaign or its impact on the mental health of health professionals. The difficulties associated with the COVID-19 vaccination campaign, due in part to the limited supply of vaccines, in part to the conflicting and changing indications for their use, and in part to the mandatory vaccination against COVID-19 in Italy, were met with a hesitant attitude toward the vaccine that made this campaign even more difficult for public health professionals. This climate of concern, coupled with mistrust, fitted into a context in which there had already been an increase in violence against health professionals for several years.

For these reasons, we decided to investigate the prevalence of workplace violence against health professionals related to the COVID-19 vaccination campaign in our region, to examine the risk and protective factors for these incidents, and to assess the impact on the victims’ mental health.

## Methods

### Study participants and study design

From March 18 to April 27, 2022, we conducted a cross-sectional study targeting all health professionals involved in the COVID-19 vaccination campaign in the Friuli-Venezia Giulia Region (Italy). The questionnaire included a total of 75 questions for two validated tests to investigate and analyze violent episodes against healthcare workers and their impact on the mental health of the workers themselves: the “Questionnaire for Workplace Violence in Healthcare Settings” (WPV) by Kumari et al. (18) and the Italian version of the “Impact of Event Scale – Revised” also known as IES-R (19, 20). The WPV questionnaire contains questions on five areas: forms of violence, impact of violent incidents, incident reporting, mitigation strategies, and risk factors. The questionnaire was translated into Italian by the research group according to the guidelines of WHO (21). The steps were: (1) independent translation of the questionnaire from English into Italian by two bilingual physicians and experts in care safety terminology and incident reporting; (2) revision of the Italian version by three experts in care safety and clinical risk management (physician, nurse, psychologist; two women and one man) who pointed out inappropriate words, phrases, or expressions and inconsistencies in the translation from English into Italian. These suggestions and proposed changes were incorporated into a revised version; (3) the revised version was back-translated into English by a bilingual person who was not involved in the previous steps; (4) this back-translated version was then compared with the original English version by two expert physicians. Particular attention was paid not to literal translation but to conceptual and cultural equivalence, as suggested by the WHO guidelines. (5) Finally, the resulting questionnaire was presented to some health professionals who might represent the population under study, in order to check in detail the understanding of each question. At this stage, the testers could point out unclear terms and suggest possible modifications to improve the understanding of the questionnaire, taking into account the objectives of the questions and the instrument. The IES-R is a well-known instrument designed for measuring symptoms of post-traumatic stress disorder (PTSD) that has been devised according to DSM-IV criteria. It is a brief, east-to-use self-report questionnaire used for repeated measures over time to monitor progress and is best used for recent and specific traumatic events. In the present work, the validated Italian version was used (22).

Sociodemographic variables such as age, sex, marital status (single, cohabiting or married with children, cohabiting or married without children, separated/divorced/widowed), profession (physician, medical resident, specialist, nurse, health assistant, auxiliary staff, other), level of education (lower secondary, upper secondary, bachelor’s degree-3 or 5/6 years, doctorate, master’s, other), area of usual work (intensive and emergency care, surgery, medicine, maternal and child, diagnostic imaging, laboratory, mental health, public health, primary care, general practitioner, recent graduate, other), years of work experience, role in COVID-19 immunization campaign (physician, administrator, front office, back office, session leader, other), previous experience with immunization services was also recorded. The full text of the questionnaire is included in Additional file 1.

The invitation to complete the questionnaire was sent by e-mail to all health professionals involved in the vaccination campaign in Friuli Venezia Giulia in the designated public vaccination centers. Health professionals who vaccinated only inpatients during hospitalization were not included; the survey was not addressed to pharmacists, since they did not administer vaccines in the Friuli Venezia Giulia region at the time of the survey. The email contained a redirection link to complete the online questionnaire on the EUSurvey platform. This platform is supported by the European Commission and can be used by researchers free of charge; the survey was conducted in full compliance with the data protection regulations currently in force at European Union level (EU-GDPR). The invitation to participate was accompanied by a description of the reasons for the study and its objectives; it was clearly stated that participation was voluntary and free of charge. The questionnaire was completely anonymous; it was not possible in any way to identify the individual participant. This survey was not part of any national or international research on the subject. Subjects who participated in the study gave their consent to the use of the data collected by completing the questionnaire. Participants were specifically asked to complete the questionnaire about their experience within the COVID-19 immunization campaign. At the end of the questionnaire, participants who wished to discuss or elaborate on their experiences of violence episodes they had experienced were given a contact person/service for psychological support.

Reading of responses, collection in a special database, and subsequent data analysis were limited to the research group. The data were managed in aggregate form, and it was not possible in any way to track the responses of individual participants. Considering that the percentage of health professionals involved in an episode of violence (threats, harassment, verbal and physical assault) is 40% according to a recent Italian survey conducted by INAIL (Italian National Institute for Insurance against Occupational Accidents) (23), it was necessary to analyze 193 questionnaires to obtain an interval estimate (95% IC) with an accuracy of 7%. The study was approved by the Unique Regional Ethical Committee of Friuli Venezia Giulia (Italy).

### Analysis of the data

Descriptive analyses were conducted to characterize the population participating in the study. Frequencies and percentages for categorical variables and means and standard deviations for continuous variables were calculated. For responses to the items of the “Questionnaire for Workplace Violence in Healthcare Settings” all responses on the 3- and 5-point Likert scales that indicated some level of agreement (moderate to strong) were scored as “agreeing responses” and those that indicated a level of disagreement “neutral” or “disagreeing” were scored as “disagreeing responses,” and then the difference was tested with a Chi-square test. The 5-point Likert scale was used to score the “Impact of Event Scale – Revised.” Results were analyzed according to Cramer et al. (20), including the three main subpatterns of avoidance, intrusiveness, and hyperarousal. We tested the normality of the distribution with the Sahpiro-Wilk test and then used parametric (t-Student) and nonparametric tests (Friedman) to compare the variables. A value of *p* < of 0.05 was considered statistically significant. Data were analyzed using IBM SPSS Statistics for Windows, version 20.0 software (Armonk, NY: IBM Corp.).

## Results

We collected 200 questionnaires, 144 (72.0%) from women, 53 (26.5%) from men, and 3 (1.5%) from individuals who preferred not to provide this information. The mean age of respondents was 46.7 ± 11.5 years, 45.8 ± 11.4 years for women and 49.6 ± 18.1 years for men. The majority of respondents were nurses (107, 53.5%), followed by physicians (71, 35.5%) and other health professionals (22, 11.0%). Key characteristics of participants, stratified by professional profile, are shown in Table 1.

**Table 1:** Participants’ characteristics.

Variable		Professional profile		
		Physician *n*. 71	Nurse *n*. 107	Other *n*. 22
Age (Mean ± DS)		45.6 ± 18.5	47.9 ± 9.8	44.7 ± 10.5
		*n* (%)	*n* (%)	*n* (%)
Sex	Women	32 (45.1)	92 (86.0)	20 (90.9)
	Men	38 (53.5)	13 (12.1)	2 (9.1)
	Missing	1 (1.4)	2 (1.9)	–
Years of service	Less than 20	44 (62.0)	35 (32.7)	13 (59.1)
	20 or more	27 (38.0)	72 (67.3)	9 (40.9)
Role in the campaign	Administrator or session leader	65 (91.5)	77 (72.0)	3 (13.6)
	Support staff	6 (8.5)	30 (28.0)	19 (86.4)
Area of usual work	Clinical and surgical care	32 (45.1)	34 (31.8)	9 (40.9)
	Directional, public health and diagnostic	20 (28.2)	17 (15.9)	9 (40.9)
	Primary care	6 (8.4)	32 (29.9)	2 (9.1)
	Emergency and intensive care	6 (8.4)	21 (19.6)	2 (9.1)
	Other	7 (9.9)	3 (2.8)	0 (0.0)

Overall, 93 (46.5%) of the 200 respondents reported being the victim of a violent act during their duty within COVID-19 immunization campaign; of these, seven described a physically violent act and 60 described a verbally violent act, and 26 did not provide information that would have been useful in determining the type of violent act. In 33 subjects (35.5%), the IES score indicated the presence of a possible post-traumatic stress reaction. Table 2 summarizes the main characteristics of health professionals stratified by the presence of a violent episode and the presence of an IES score ≥ 33; the only characteristic that had a statistically significant effect on the presence of violent episode was a higher study title. For the presence of possible post-traumatic stress (PTS) symptoms, campaign role and service area were the characteristics that had an influence; the risk of PTS was higher in vaccine administrators and session leaders, and lower in professionals who normally work in emergency care or intensive care units.

**Table 2:** Characteristics of health professionals involved in violent episodes and who developed post-traumatic stress symptoms.

	Participants *N* = 200	Episode of violence *N* = 93	*p*-value	Post- traumatic stress symptoms *N* = 33	*p*-value
	*N* (%)	*N* (%)		*N* (%)	
**Professional profile**					
Other	22 (11.0)	8 (8.6)	–	4 (12.1)	–
Nurse	107 (53.5)	47 (50.5)		19 (57.6)	
Physician	71 (35.5)	38 (40.9)		10 (30.3)	
**Sex**					
Women	144 (72.0)	64 (68.8)	–	23 (69.7)	–
Men	53 (26.5)	27 (29.0)		8 (24.2)	
Missing	3 (1.5)	2 (2.2)		2 (2.1)	
**Age group (years)**					
20–40	70 (35.0)	33 (35.5)	–	13 (39.4)	–
41–60	103 (51.5)	47 (50.5)		18 (54.5)	
61 or more	27 (13.5)	13 (14.0)		2 (2.1)	
**Years of service**					
Less than 20	92 (46.0)	44 (47.3)	–	17 (51.5)	–
20 or more	108 (54.0)	49 (52.7)		16 (48.5)	
**Education**					
Bachelor degree or lower	77 (38.5)	28 (30.1)	<0.05	10 (30.3)	–
Master’s degree or higher	123 (61.5)	65 (69.9)		23 (69.7)	
**Role in the campaign**					
Administrator or session leader	145 (72.5)	67 (72.0)	-	18 (54.5)	<0.05
Support staff	55 (27.5)	26 (28.0)		15 (45.4)	
**Area of service**					
Directional, public health and diagnostic	46 (23.0)	22 (26.7)	-	10 (30.3)	<0.05
Emergency care and ICU	29 (14.5)	11 (11.8)		3 (9.1)	
Clinical and surgical care	75 (37.5)	38 (40.9)		8 (24.2)	
Primary care	40 (20.0)	16 (17.2)		10 (30.3)	
Other	10 (5.0)	6 (6.4)		2 (2.1)	

The difference in IES score was not statistically significant between those who had suffered physical violence (30.14 ± 15.39) and those who had suffered verbal violence (27.30 ± 16.18).

When the scores for each of the three parts of the IES were analyzed, the mean score for avoidance was 1.15 ± 0.66, for intrusiveness was 1.31 ± 0.89, and for hyperarousal was 1.45 ± 0.90, with a statistically significant difference (*p* < 0.01). Analysis of these data for each professional role yielded IES scores of avoidance 1.03 ± 0.55, intrusiveness 1.20 ± 0.77, and hyperarousal 1.37 ± 0.81 for physicians; avoidance 1.26 ± 0.71, intrusiveness 1.37 ± 0.97, and hyperarousal 1.52 ± 0.99 for nurses; and avoidance 1.00 ± 0.79, intrusiveness 1.39 ± 0.94, and hyperarousal 1.40 ± 0.88 for the other health professions.

Table 3 summarizes the prevalence of agreeing responses of the total surveyed population to the questions about workplace violence prevention and support activities for workers. For all questions, there were no statistical differences in the prevalence of agreement among the three professional profiles. Significant differences by sex are highlighted in the table.

**Table 3:** Participants’ opinions on the impact of WPV, violence reporting behavior, strategies to mitigate violence, and risk factors according to professional profile and sex.

Item	Overall *n*. 200		Professional profile						Sex			
			Physicians *n*. 71		Nurses *n*. 107		Other *n*. 22		Female *n*. 144		Male *n*. 53	
	Disagree	Agree	Disagree	Agree	Disagree	Agree	Disagree	Agree	Disagree	Agree	Disagree	Agree
The effect of the episodes of WPV one had on the different aspects of life												
How much have the episodes of violence at your workplace affected your personal wellbeing and self-care?*	135 (67.5)	65 (32.5)	47 (66.2)	24 (33.8)	69 (64.5)	38 (35.5)	19 (86.4)	3 (13.6)	95 (66.0)	49 (34.0)	39 (73.6)	14 (26.4)
How much has your family been affected due to the episodes of violence at your workplace?**	145 (72.5)	55 (27.5)	50 (70.4)	21 (29.6)	78 (72.9)	29 (27.1)	17 (77.3)	5 (22.7)	101 (70.1)	43 (29.9)	43 (81.1)	10 (18.9)
How much has your social life been affected due to the episodes of violence at your workplace?***	148 (47.0)	52 (26.0)	56 (78.9)	15 (21.1)	75 (70.1)	32 (29.9)	17 (77.3)	5 (22.7)	103 (71.5)	41 (28.5)	44 (83.0)	9 (17.0)
How much do the episodes of violence at your workplace has affected your mental and psychological well-being (increased aggressiveness, irritability, low self-esteem, etc.)?	102 (51.0)	98 (49.0)	37 (52.1)	34 (47.9)	51 (47.7)	56 (52.3)	14 (63.6)	8 (36.4)	69 (47.6)	75 (52.1)	32 (60.4)	21 (39.6)
Reporting of incidence												
I would be comfortable in reporting the episode of violence at my workplace to competent authorities.	34 (17.0)	116 (83.0)	14 (19.7)	57 (80.3)	18 (16.8)	89 (83.2)	2 (9.1)	20 (90.9)	25 (17.4)	119 (82.6)	7 (13.2)	46 (86.8)
Extent to which these following reasons lead to under-reporting?												
Felt ashamed of reporting	104 (52.0)	96 (48.0)	39 (54.9)	32 (45.1)	58 (54.2)	49 (45.8)	7 (31.8)	15 (68.2)	70 (48.6)	74 (51.4)	32 (60.4)	21 (39.6)
A belief that no action will be taken against the perpetrator**// per sex (*p* = 0.003)**	21 (10.5)	179 (89.5)	11 (15.5)	60 (84.5)	9 (8.4)	98 (91.6)	1 (4.5)	21 (95.5)	**9 (6.3)**	**135 (93.8)**	**11 (20.8)**	**42 (79.2)**
Lack of organizational support	22 (11.0)	178 (89.0)	8 (11.3)	63 (88.7)	11 (10.3)	96 (89.7)	3 (13.6)	19 (86.4)	16 (11.1)	128 (88.9)	6 (11.3)	47 (88.7)
Lack of provision to report such incidences	45 (22.5)	155 (77.5)	13 (18.3)	58 (81.7)	26 (24.3)	81 (75.7)	6 (27.3)	16 (72.7)	34 (23.4)	110 (76.4)	11 (20.8)	42 (79.2)
The process was time-consuming	52 (26.0)	148 (74.0)	14 (19.7)	57 (80.3)	32 (29.9)	75 (70.1)	6 (27.3)	16 (72.7)	42 (29.2)	102 (70.8)	10 (18.9)	43 (81.1)
Fear that the appraisal or promotion avenues will be affected	108 (54.0)	92 (46.0)	42 (59.2)	29 (40.8)	56 (52.3)	51 (47.7)	10 (45.5)	12 (54.5)	75 (52.1)	69 (47.9)	33 (62.3)	20 (37.7)
Mitigation Strategies												
Controlling the number of attendants visiting the hospital with a patient	29 (14.5)	171 (85.5)	11 (15.5)	60 (84.5)	16 (15.0)	91 (85.0)	2 (9.1)	20 (90.9)	21 (14.6)	123 (85.4)	8 (15.1)	45 (84.9)
Educating patients and attendants about limitations of medical sciences and available infrastructure	16 (8.0)	184 (92.0)	5 (7.0)	66 (93.0)	11 (10.3)	96 (89.7)	-	22 (100)	12 (8.3)	132 (91.7)	4 (7.5)	49 (92.5)
Regular training of healthcare workers regarding soft skills (communication skills, breaking bad news, counseling skills, problem solving skills)	7 (3.5)	193 (96.5)	5 (7.0)	66 (93.0)	2 (1.9)	105 (98.1)	-	22 (100)	4 (2.8)	140 (97.2)	3 (5.7)	50 (94.3)
Self-defence training of Health care workers**// per sex (*p* < 0.001)**	50 (25.0)	150 (75.0)	23 (32.4)	48 (67.6)	24 (22.4)	83 (77.6)	3 (13.6)	19 (86.4)	**25 (17.4)**	**119 (82.6)**	**24 (45.3)**	**29 (54.7)**
Improving healthcare facilities (like doctor-patient ratio, population-bed ratio)	3 (1.5)	197 (98.5)	-	71 (100)	3 (2.8)	104 (97.2)	-	22 (100)	2 (1.4)	142 (98.6)	1 (1.9)	52 (98.1)
Improving facilities within a hospital (like availability of medicines and diagnostic tests)	33 (16.5)	167 (83.5)	10 (14.1)	61 (85.9)	20 (18.7)	87 (81.3)	3 (13.6)	19 (86.4)	21 (14.6)	123 (85.4)	12 (22.6)	41 (77.4)
Improving Infrastructure facilities (like installation of CCTVs, metal detectors, alarm system)	13 (6.5)	187 (93.5)	6 (8.5)	65 (91.5)	6 (5.6)	101 (94.4)	1 (4.5)	21 (95.5)	9 (6.3)	135 (93.8)	4 (7.5)	49 (92.5)
Active complaint redressal system	24 (12.0)	176 (88.0)	10 (14.1)	61 (85.9)	13 (12.1)	94 (87.9)	1 (4.5)	21 (95.5)	14 (9.7)	130 (90.3)	10 (18.9)	43 (81.1)
Strong legislature measures like provision of significant punishment for offenders	6 (3.0)	194 (97.0)	2 (2.8)	69 (97.2)	3 (2.8)	104 (97.2)	1 (4.5)	21 (95.5)	4 (2.8)	140 (97.2)	2 (3.8)	51 (96.2)
Unbiased media reporting	8 (4.0)	192 (96.0)	3 (4.2)	68 (95.8)	5 (4.7)	102 (95.3)	-	22 (100)	5 (3.5)	139 (96.5)	3 (5.7)	50 (94.3)
Sensitizing politicians and public figures not to give immature/negative statements regarding healthcare workers	5 (2.5)	195 (97.5)	2 (2.8)	69 (97.2)	3 (2.8)	104 (97.2)	-	22 (100)	4 (2.8)	140 (97.2)	1 (1.9)	52 (98.1)
Peaceful working climate (missing = 1)	-	199 (100)	-	70 (100)	-	107 (100)	-	22 (100)	-	143 (100)	-	53 (100)
Availability of support from colleagues (missing = 1)	1 (0.5)	198 (99.5)	1 (1.4)	69 (98.6)	-	107 (100)	-	22 (100)	-	143 (100)	1 (1.9)	52 (98.1)
Risk factors related to incidents of Workplace violence												
Unrealistic expectations of patients/attendants	-	200 (100)	-	71 (100)	-	107 (100)	-	22 (100)	-	144 (100)	-	53 (100)
Inappropriate knowledge about the disease/health condition	6 (3.0)	194 (97.0)	3 (4.2)	68 (95.8)	3 (2.8)	104 (97.2)	-	22 (100)	6 (4.2)	138 (95.8)	-	53 (100)
Poor communication skills	6 (3.0)	194 (97.0)	2 (2.8)	69 (97.2)	4 (3.7)	103 (96.3)	-	22 (100)	3 (2.1)	141 (97.9)	3 (5.7)	50 (94.3)
Lack of resources (equipment and medicines, doctor-patient ratio)**// per sex (*p* = 0.026)**	24 (12.0)	176 (88.0)	13 (18.3)	58 (81.7)	10 (9.3)	97 (90.7)	1 (4.5)	21 (95.5)	**13 (9.0)**	**131 (91.0)**	**11 (20.8)**	**42 (79.2)**
Overcrowding	7 (3.5)	193 (96.5)	3 (4.2)	68 (95.8)	4 (3.7)	103 (96.3)	-	22 (100)	5 (3.5)	139 (96.5)	2 (3.8)	51 (96.2)
Long waiting time	-	200 (100)	-	71 (100)	-	107 (100)		22 (100)	-	144 (100)	-	53 (100)
Inadequate security arrangements**// per sex (*p* = 0.020)**	18 (9.0)	182 (91.0)	8 (11.3)	63 (88.7)	9 (8.4)	98 (91.6)	1 (4.5)	21 (95.5)	**9 (6.3)**	**135 (93.8)**	**9 (17.0)**	**44 (83.0)**
Inadequate action on receiving complaints of WPV	1 (0.5)	199 (99.5)	1 (1.4)	70 (98.6)	-	107 (100)	-	22 (100)	-	144 (100)	1 (1.9)	52 (98.1)
Lack of respect for the authority of doctors/healthcare workers	3 (1.5)	197 (98.5)	2 (2.8)	69 (97.2)	1 (0.9)	106 (99.1)	-	22 (100)	2 (1.4)	142 (98.6)	1 (1.9)	52 (98.1)
Negative and inappropriate media reporting	4 (2.0)	196 (98.0)	2 (2.8)	69 (97.2)	2 (1.9)	105 (98.1)	-	22 (100)	1 (0.7)	143 (99.3)	3 (5.7)	50 (94.3)
Lack of the provision of harsh punishment for aggressors/offenders	12 (6.0)	188 (94.0)	5 (7.0)	66 (93.0)	6 (5.6)	101 (94.4)	1 (4.5)	21 (95.5)	6 (4.2)	138 (95.8)	6 (11.3)	47 (88.7)
Lack of redressal system**// per sex (*p* = 0.024)**	21 (10.5)	179 (89.5)	11 (15.5)	60 (84.5)	9 (8.4)	98 (91.6)	1 (4.5)	21 (95.5)	**11 (7.6)**	**133 (92.4)**	**10 (18.9)**	**43 (81.1)**

*Personal wellbeing and self-care include activities such as sleep schedule, eating pattern, fitness, grooming, dressing etc.; **Family life is defined as the routine interactions and activities that a family have together especially with the members who live together with parents, spouse, children; ***Social life is defined as the part of a person’s time spent doing enjoyable things with others like friends, colleagues or people living in the society other than close family member. In bold are reported statistically significant differences among values.

## Discussion

The aim of our study was to investigate the prevalence of workplace violence against health professionals involved in the COVID -19 vaccination campaign in our region, to identify the risk and protective factors for these incidents, and to assess their impact on the mental health of the victims.

We found that 46.5% of health professionals who participated in our regional COVID -19 vaccination campaign reported being a victim of a physical or verbal act of violence in the workplace. In general, half of health professionals reported that the consequences of workplace violence affected their mental and psychological well-being, and about one-third reported that these consequences also affected their family and social life. The likelihood of reporting workplace violence was evenly distributed among health professionals, with the exception of those with higher levels of education, who were more likely to report such incidents. Although most incidents of workplace violence were verbal in nature, more than one-third of victims developed post-traumatic stress symptoms. The incidence of post-traumatic stress symptoms was higher among frontline professionals, who were likely to be more exposed to the stress, anger, and frustration of citizens who visited the immunization centre, than among those who supported campaign activities from the back office. In contrast, professionals who normally work in emergency situations reported lower levels of stress. This could be related to some skills and competencies they acquired in their professional context and background, or to some coping strategies they developed personally or with the support of psychologists supervising their units. However, they may also be more accustomed to such situations, which may have led to some underreporting.

The impact of COVID-19 on the mental health of the general population is widely recognized (12), and has been called the perfect storm for mental health by some colleagues (13). The importance and burden of pandemics on the mental health of healthcare workers is also not new to the scientific community. For example, the systematic review and meta-analysis by Hills et al. estimated the prevalence of post-traumatic stress disorder at 21.7%, anxiety at 16.1%, major depressive disorder at 13.4%, and acute stress disorder at 7.4% (24). However, starting from a stable trend of WPV reported in Italy in the years preceding the pandemic (25), an increase in workplace violence, mostly by patients (type II), and especially in emergency departments, was observed in the years of COVID-19 (6, 11, 26). Although some authors reported a higher prevalence of workplace violence in men (7, 27), this was not the case in our setting, which seemed to confirm the absence of sex differences reported in an Italian analysis before COVID (25). Other findings related to a higher prevalence in older (7) or younger (27) health professionals were not confirmed by our data.

The role of WPV’s added psychological trauma in pandemic fatigue and its contribution to decreased job satisfaction (7), the development of mental illness (6), decreased empathy skills (28), burnout, resulting turnover intent (29), and the unprecedented exodus of public health professionals we are currently experiencing has been explored but requires further research and investment to address this critical issue (11). In any case, given the impact on the mental health and well-being of the victim, it is important to recognize verbal abuse as a form of workplace violence that should be reported and addressed (2). This is even more important when considering the potential impact of these incidents on patient access and patient safety, which are fundamental to health care (30), and considering that the impact on the family and social relationships of health professional involved in a violent incident may exacerbate the situation for the victim (6).

Our data seem to confirm that frontline health professionals and especially those working in highly politicized settings, as described by Kuhlmann et al. (17), as well as preventive health services, such as COVID-19 immunization services, are among the main target groups of WPV.

The actual extent of this phenomenon still seems to be underestimated, and health professionals cited lack of confidence in an effective reporting system (i.e., lack of rules for reporting such incidents, time-consuming process) and lack of confidence in the administration and action taken (i.e., belief that no action will be taken against the perpetrator, lack of organizational support) as possible reasons for this attitude. Indeed, in many cases, participants reported that the risk for violent incidents was higher when effective communication was absent or inadequate. Just as continuous training in so-called hard skills is mandatory for health professionals, it might be useful for healthcare institutions to organize structural courses to improve soft skills, which can be useful not only in private life but also in everyday work. Indeed, these skills may have played an important role in preventing the development of post-traumatic stress reactions among the emergency specialists in our sample. Soft skills that should be learned by health professionals for this purpose certainly include effective communication, but teamwork and conflict management would also likely help mitigate many of the scenarios found in our study. Regarding training, the fact that a fairly large number of health professionals indicated that they would feel safer if they had taken a self-help course can be seen both as a purpose for a specific training course and as evidence of distrust in the healthcare organization and its ability to address the problem in the future. Although many respondents indicated that they would feel comfortable reporting incidents of violence, we cannot ignore the fact that several participants indicated that this was not the case. This may be primarily because they believe it is unnecessary to report because there was no uniform official reporting system at the time of the study, but also because they believe that no action is taken against the perpetrators of violence.

Most of the mitigation strategies proposed in the questionnaire met with the respondents’ agreement, with nine of the 13 items receiving a general approval of over 90%. The most important ones can be divided into the following groups: (1) relationships – such as the existence of a peaceful working climate (100%), the availability of supportive colleagues (99.5%); (2) organization – such as the improvement of health facilities in terms of doctor-to-patient and population-to-patient ratios (98.5%), the management or avoidance of overcrowding (96.5%), and the availability of technological equipment (e.g., video surveillance, metal detectors, alarm systems; 93.5%); and (3) communication, both political (97.5%) and media (96.0%), but also taking into account the training of individual health professionals in soft skills (96.5%). Other authors suggested classifying the same and other risk/protective factors according to their affiliation with the workplace and policy, patient, physicians, physician-patient relationship, and sociocultural aspects (6). In any case, effective communication is undoubtedly considered the first step to reduce the incidence of aggression by patients, improve the experience of healthcare staff in dealing with such incidents, and help them maintain their psychological well-being in the long term (31).

Although stigmatization of health professionals during the pandemic was reported as a common phenomenon in low-income countries (32), the expression of public anguish, likely resulting from the negative emotional impact on the general population due to restrictions on social and economic activities and disruption of services (26), appears to be consistently common in middle- and higher-income countries (32). This phenomenon of anger and violence against health care workers during pandemics has been analysed by colleagues who noted a pattern that seems to be repeated throughout history regardless of the left or right orientation of government (33). Indeed, the occurrence of the II WPV type is a negative trigger for the quality of the trust relationship between health professionals and patients, as well as a sensitive thermometer of psychosocial risk factors. In addition, the problem of patients’ unrealistic expectations of science and medicine emerges from the analysis of several questions about potential containment strategies and WPV risk factors. Nonetheless, the issue of public trust in the healthcare system, including all levels from frontline health workers to their leaders/managers, and the government providing resources, emerges in the backyard and may have played a role, especially in the context of a massive vaccination campaign such as that conducted for COVID -19. Conspiracy beliefs have been linked to intentions of violence, showing that such theories are not harmless. Their association with communication limited to one’s own echo chamber, which has been observed with other topics of public interest such as climate change, genetically modified organisms, and the origin of pathogens, can lead individuals to make risky health decisions and greatly endanger public health at the population level (34).

Although health professionals are expected to care for patients, we should always remember that they may suffer because of their work. Indeed, the suicide rate among healthcare workers because of WPV suffered or other management problems is not known and should be further investigated. We agree with colleagues who say that health care workers, like all other workers, have a right to safety in the workplace (2, 6). Therefore, we believe that employers and governments have duties to their employees and should adhere to some sort of ethical code by ensuring the care of their employees, investigating and sanctioning health care violence (4), and protecting and promoting the well-being of health care workers (5). With regard to workplace violence, a zero-tolerance policy should be developed, and legal action taken against perpetrators (3, 7). In light of recent statements by the Italian Ministry of Health (35), some changes seem to be emerging, but the actual implementation of this commitment will be evaluated soon. In the meantime, institutions and colleagues are taking steps toward a safer work environment for health professionals, for example, in pediatric clinics (36), even using simulations based on improvisational theater (37). Much work remains to be done in this area to develop structured strategies. Possible interventions suggested in the scientific literature include actions at the organizational and individual levels, with training and education on violence prevention, attention to at-risk patients, increased security measures, development of safety standards in health care facilities, and timely response after acts of violence (6). Strategies that include both prevention of episodes of violence and management of violence that has already occurred must be implemented in parallel (31). In addition, specifically in the case of vaccination, the government and public health organizations should work to ensure that the vaccination process remains apolitical and counter misinformation that could fuel anger or fear (38).

### Limitations and strengths

This study has several limitations that must be considered in order to better interpret and utilize our results. First, our data refer only to the Friuli-Venezia Giulia region, so the generalizability of the results at the national or international level cannot be guaranteed, also in view of the different burden of vaccine hesitancy and the resulting COVID-19 vaccination adherence. Furthermore, because of the cross-sectional nature of the study, a causal relationship cannot be inferred. Second, the data were collected using an online questionnaire, so participants were not assisted in answering the questions, which may have introduced bias in the number of reported acts of WPV, particularly underreporting of verbal violence, which is often not considered an act of violence. In addition, some recall bias may have occurred because we asked participants to report episodes of violence that occurred during their service as part of the vaccination campaign that began in Europe in late 2020. Third, we do not have information from those healthcare professionals who were involved in the vaccination campaign but did not participate in our survey despite being invited, so we cannot rule out selection bias. Finally, because of the anonymous nature of the survey, we could not calculate the potential exposure to workplace violence for each participant in the COVID-19 vaccination campaign. However, our strengths include the use of two validated instruments to measure the occurrence or impact of workplace violence. In addition, we chose to include all professional groups involved in the vaccination campaign at the regional level to obtain a multiprofessional perspective on the phenomenon.

## Conclusion

One third of the health professionals involved in the COVID-19 immunization campaign reported that their mental health and well-being were affected by violence perpetrated during their service. More attention should be paid to public health professionals who deal with politicized and much debated issues such as immunization. Nevertheless, a more structured and multidisciplinary approach to the problem needs to be adopted, addressing all aspects, including legal and psychological support, information, education and training, reporting system, and quality improvement.

## Data availability statement

The original contributions presented in the study are included in the article/supplementary material, further inquiries can be directed to the corresponding author/s.

## Ethics statement

The studies involving humans were approved by the Unique Regional Ethical Committee of Friuli Venezia Giulia (Italy). The studies were conducted in accordance with the local legislation and institutional requirements. The participants provided their written informed consent to participate in this study.

## Author contributions

LB: Conceptualization, Data curation, Investigation, Methodology, Project administration, Writing – original draft. ES: Conceptualization, Investigation, Methodology, Writing – review & editing. TL: Conceptualization, Data curation, Writing – review & editing. FrF: Conceptualization, Investigation, Methodology, Writing – review & editing. FC: Investigation, Methodology, Writing – review & editing. PZ: Conceptualization, Writing – review & editing. FeF: Investigation, Methodology, Writing – review & editing. EC: Data curation, Writing – review & editing. BP: Data curation, Writing – review & editing. RC: Investigation, Methodology, Writing – review & editing. LA: Conceptualization, Formal analysis, Investigation, Methodology, Writing – review & editing.

## Funding

The author(s) declare that no financial support was received for the research, authorship, and/or publication of this article.

## Conflict of interest

The authors declare that the research was conducted in the absence of any commercial or financial relationships that could be construed as a potential conflict of interest.

## Publisher’s note

All claims expressed in this article are solely those of the authors and do not necessarily represent those of their affiliated organizations, or those of the publisher, the editors and the reviewers. Any product that may be evaluated in this article, or claim that may be made by its manufacturer, is not guaranteed or endorsed by the publisher.
